# Supplementary material for: Molecular characterization of a marine turtle tumor epizootic, profiling external, internal and postsurgical regrowth tumors
Source: Commun Biol. 2021 Feb 1;4:152. doi: 10.1038/s42003-021-01656-7 (PMC7851172; doi:10.1038/s42003-021-01656-7)
Supplement: Supplementary file 2 — Supplementary Information [file 42003_2021_1656_MOESM2_ESM.pdf]

SUPPLEMENTARY INFORMATION

FOR

Molecular characterization of a marine turtle tumor epizootic, profiling external, internal and post-surgical regrowth tumors

Kelsey Yetsko[1^], Jessica A. Farrell[1,2^], Nicholas B. Blackburn[3,4], Liam Whitmore[1,5], Maximilian R. Stammnitz[6], Jenny Whilde[1], Catherine B. Eastman[1], Devon Rollinson Ramia[1], Rachel Thomas[1], Aleksandar Krstic[7], Paul Linser[1], Simon Creer[8], Gary Carvalho[8], Mariana A. Devlin[9], Nina Nahvi[9], Ana Cristina Leandro[3,4], Thomas W. deMaar[10], Brooke Burkhalter[1], Elizabeth P. Murchison[6], Christine Schnitzler[1,2] and David J. Duffy[1,2,5,7,8]*.

[1] The Whitney Laboratory for Marine Bioscience and Sea Turtle Hospital, University of Florida, St. Augustine, Florida 32080, USA.
[2] Department of Biology, University of Florida, Gainesville, Florida, 32611, USA.
[3] Department of Human Genetics, School of Medicine, University of Texas Rio Grande Valley, Brownsville, Texas, USA.
[4] South Texas Diabetes and Obesity Institute, School of Medicine, University of Texas Rio Grande Valley, Brownsville, Texas, USA.
[5] Department of Biological Sciences, School of Natural Sciences, Faculty of Science and Engineering, University of Limerick, Limerick, Ireland.
[6] Transmissible Cancer Group, Department of Veterinary Medicine, University of Cambridge, Cambridge CB3 0ES, UK.
[7] Systems Biology Ireland & Precision Oncology Ireland, School of Medicine, University College Dublin, Belfield, Dublin 4, Ireland.
[8] Molecular Ecology and Fisheries Genetics Laboratory, School of Biological Sciences, Bangor University, Bangor, Gwynedd LL57 2UW, UK.
[9] Sea Turtle Inc., South Padre Island, Texas, USA.
[10] Gladys Porter Zoo, Brownsville, Texas, USA.

[^]These authors contributed equally to this work

*****Correspondence to: David J. Duffy**.** Email: duffy@whitney.ufl.edu

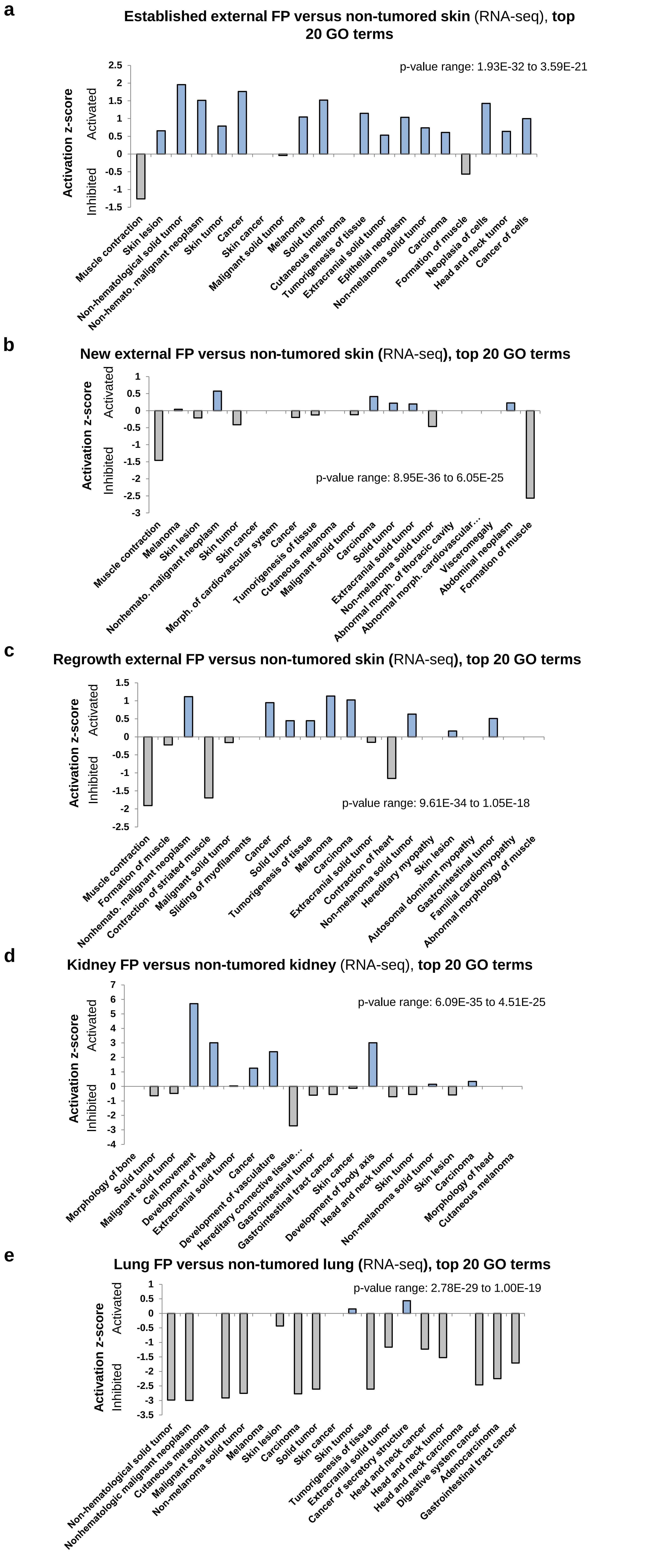

Supplemental Figure 1. Additional activation/inhibition z-scores of the top 20 disease-associated GO terms associated with transcripts differentially expressed in different types of fibropapillomatosis tumors (RNA-seq), as detected by IPA, ranked by *P*-value (calculated by right-tailed Fisher's Exact Test, with Benjamini-Hochberg correction). **a)** Established external FP versus non-tumored skin. A total of 689 of the significant DEs were recognized by IPA and used in the analysis. **b)** New external FP versus non-tumored skin. A total of 698 of the significant DEs were recognized by IPA and used in the analysis. **c)** Regrowth external FP versus non-tumored skin. A total of 507 of the significant DEs were recognized by IPA and used in the analysis. **d)** Kidney FP versus non-tumored kidney tissue. A total of 618 of the significant DEs were recognized by IPA and used in the analysis. **e)** Lung FP versus non-tumored lung tissue. A total of 653 of the significant DEs were recognized by IPA and used in the analysis.

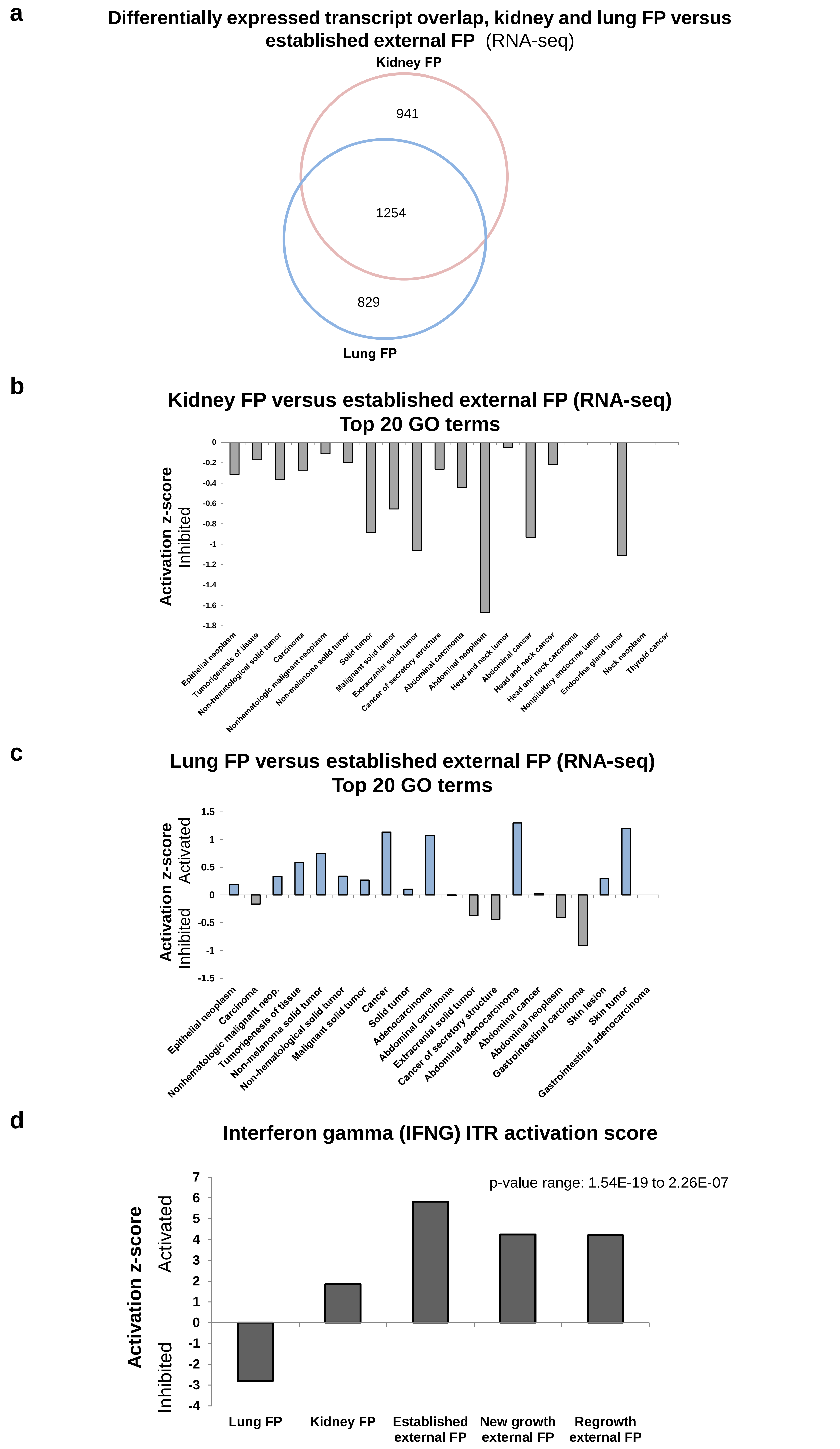

Supplemental Figure 2. Additional fibropapillomatosis transcriptomic comparisons. **a)** Overlap of transcripts from RNA-seq data significantly differentially expressed (DE) (as called by DESeq2) in fibropapillomatosis between either kidney FP compared to established external FP, or lung FP compared to established external FP. Transcripts were considered significant if passing the following cut-offs: adjusted *P*-value of < 0.05 and $\log_2$ fold change of > 2 and < -2. **b, c)** Activation/inhibition z-scores of the top 20 disease-associated gene ontology (GO) terms associated with transcripts differentially expressed in different types of fibropapillomatosis tumors (RNA-seq), as detected by IPA, ranked by *P*-value (calculated by right-tailed Fisher's Exact Test, with Benjamini-Hochberg correction). **b)** Kidney FP versus established external FP tumors. **c)** Lung FP versus established external FP tumors. **d)** Activation z-scores of the Interferon gamma (IFNG) inferred transcriptional regulator (ITR) associated with transcripts differentially expressed across the five different types of fibropapillomatosis tumors, when compared to their respective non-tumor tissue sources.

a

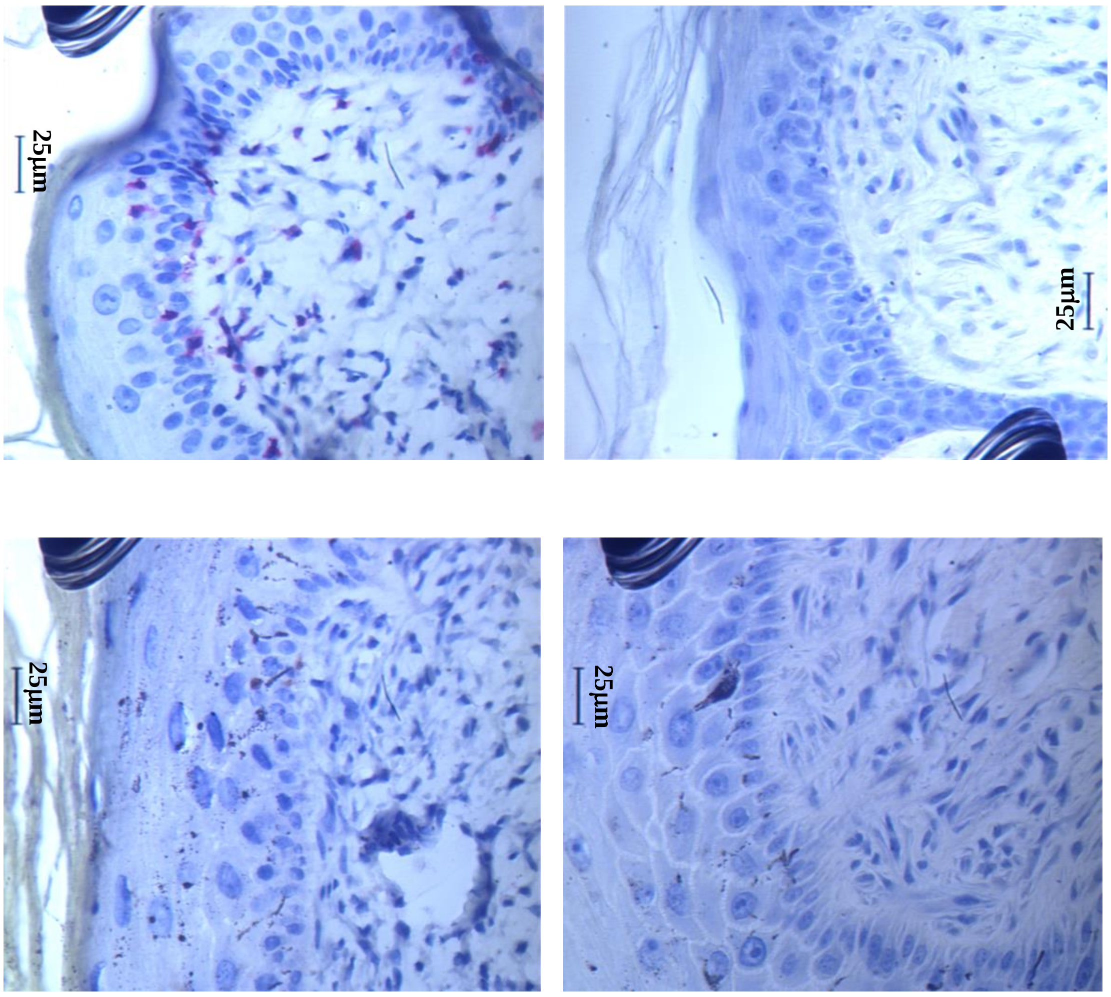

b

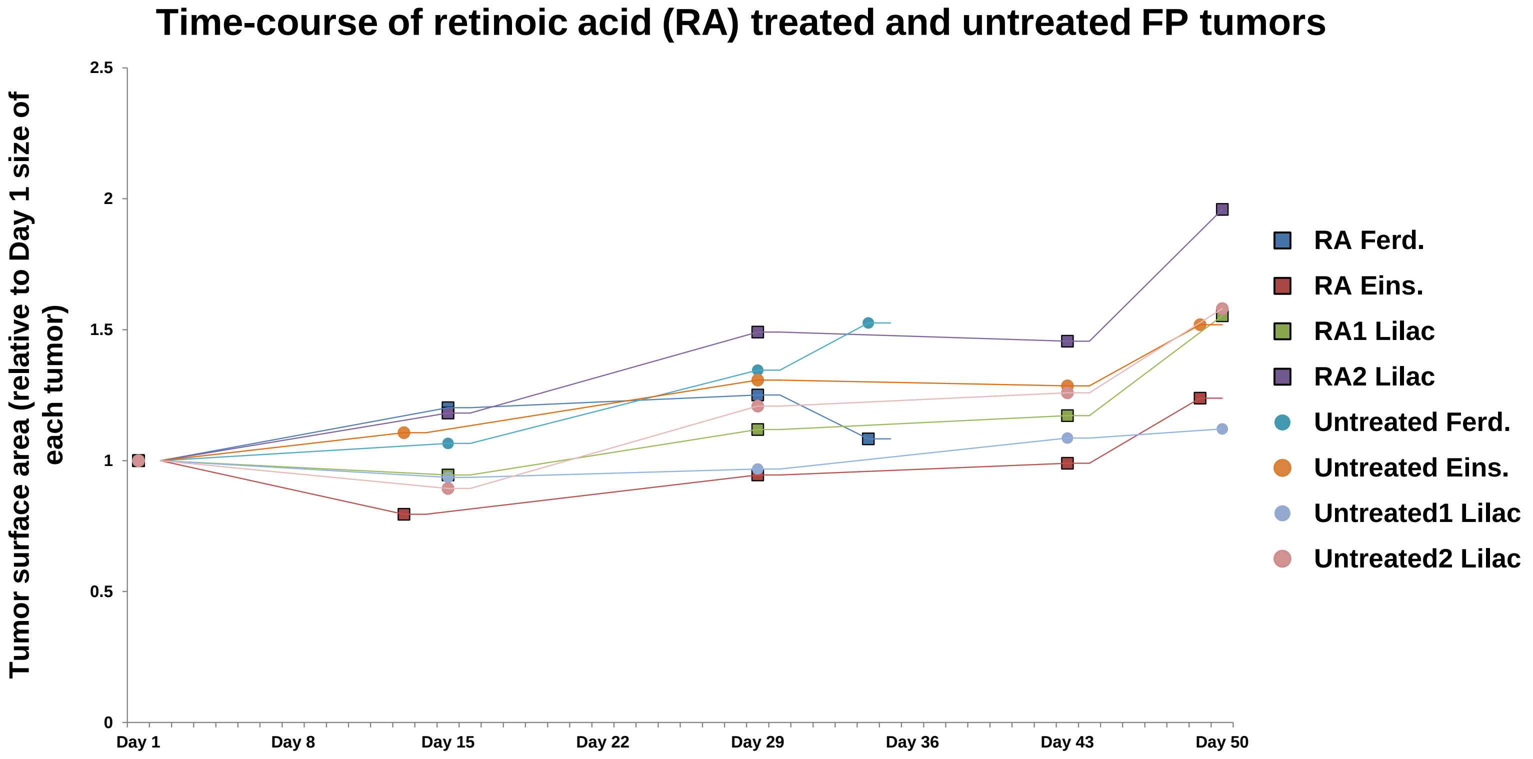

Supplemental Figure 3. Additional CD3 staining and RA treatment tumor growth profiling. **a)** CD3 antibody-based staining (red/purple) of T lymphocyte infiltration in an established external tumor and regrowth external tumor, nuclei are counterstained with Hematoxylin (blue staining). **b)** Time-course of relative tumor growth profiles of retinoic acid (RA, Tretinoin cream 0.1%) treated and untreated fibropapillomatosis tumors. Profiles for eight tumors across three individual *C. mydas* patients are shown. Duration of treatment was under veterinary determination. Patient 'name' abbreviations, Ferd., 'Ferdinand' (07-2018-Cm), Eins., 'Einstein' (28-2018-Cm) and 'Lilac' (25-2018-Cm). Tumor growth is relative to the size of each individual tumor before treatment, i.e. Day 1.

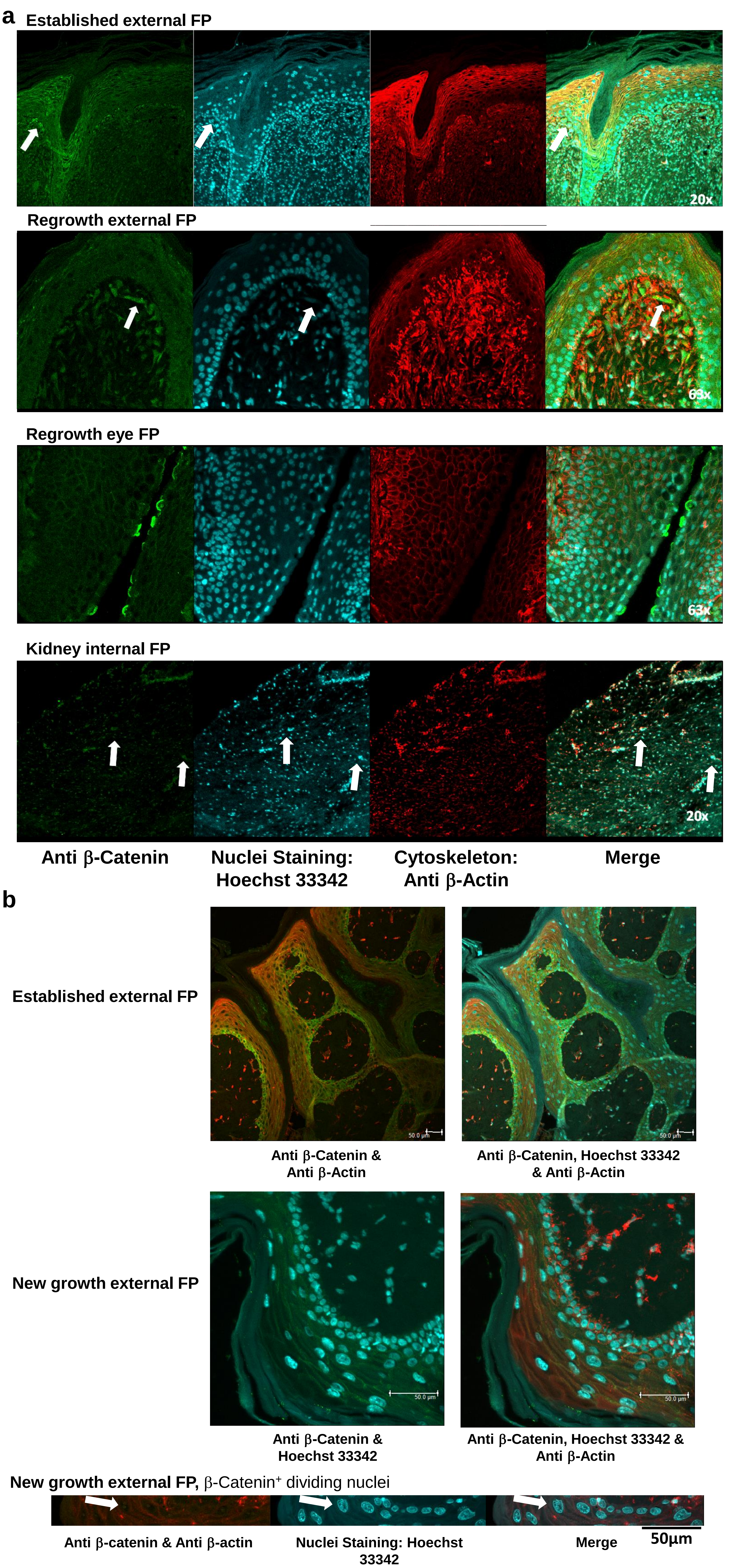

Supplemental Figure 4. Additional fibropapillomatosis tumor immunohistochemistry. **a)** Antibody-based immunohistochemistry of established external, regrowth external, regrowth eye external and kidney internal tumor tissue. Tissue sections are stained for β-catenin (anti- β-catenin antibody) and counter stained with Hoechst 33342 to visualize nuclei and Anti β-actin. Selected cells with nuclear (activated) β -catenin staining are indicated by white arrows. **b)** Established external and new growth tumor sections, stained for β-catenin (anti- β-catenin antibody) and counter stained for Hoechst 33342 and Anti β-actin, highlighting the membrane localization of β-catenin in epidermal tumor cells, top, and dividing nuclei localization, bottom.

a

Established external FP versus non-tumored skin (RNA-seq)
Top 200 ITRs

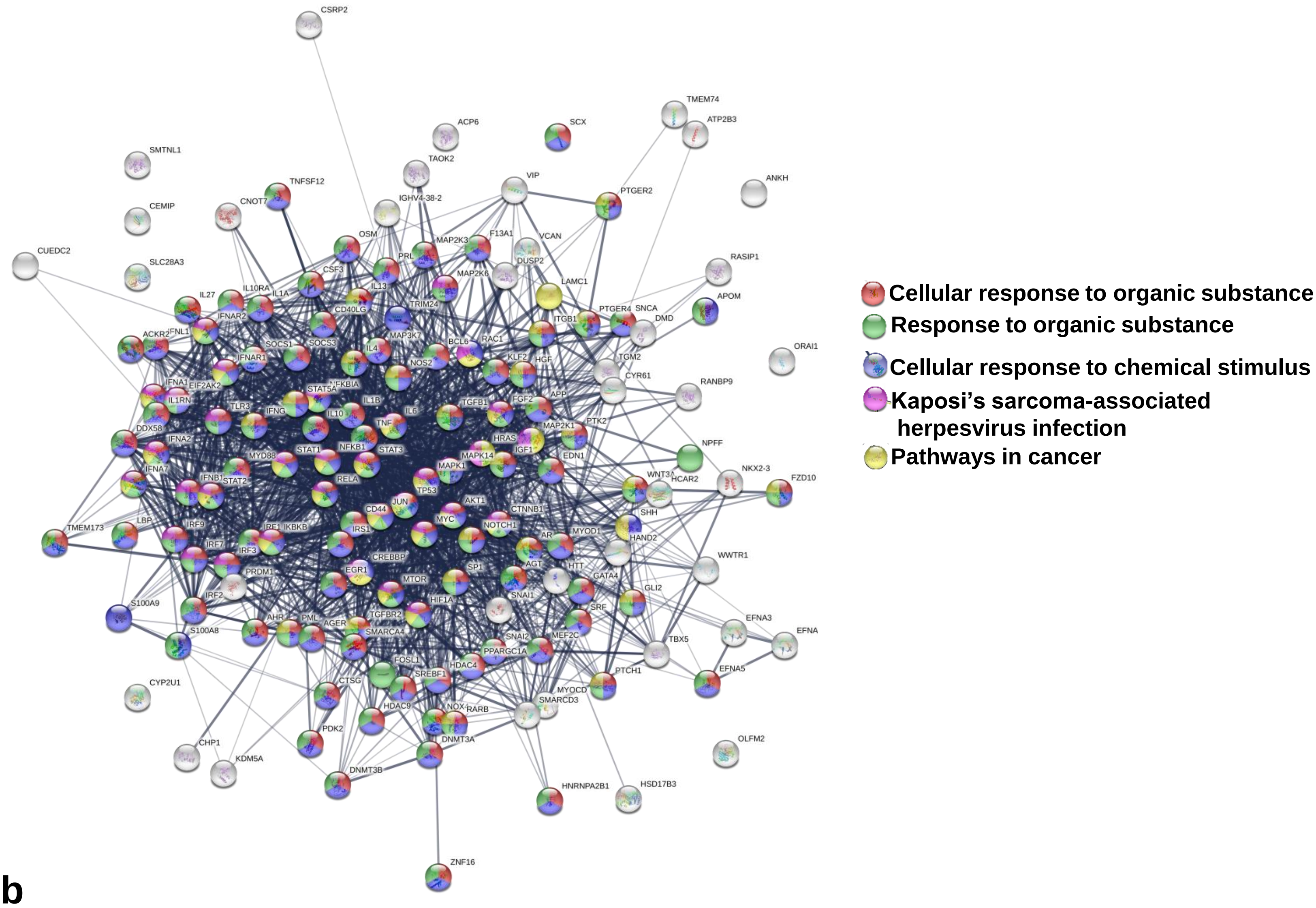

b

Lung FP versus non-tumored skin (RNA-seq)
Top 200 ITRs

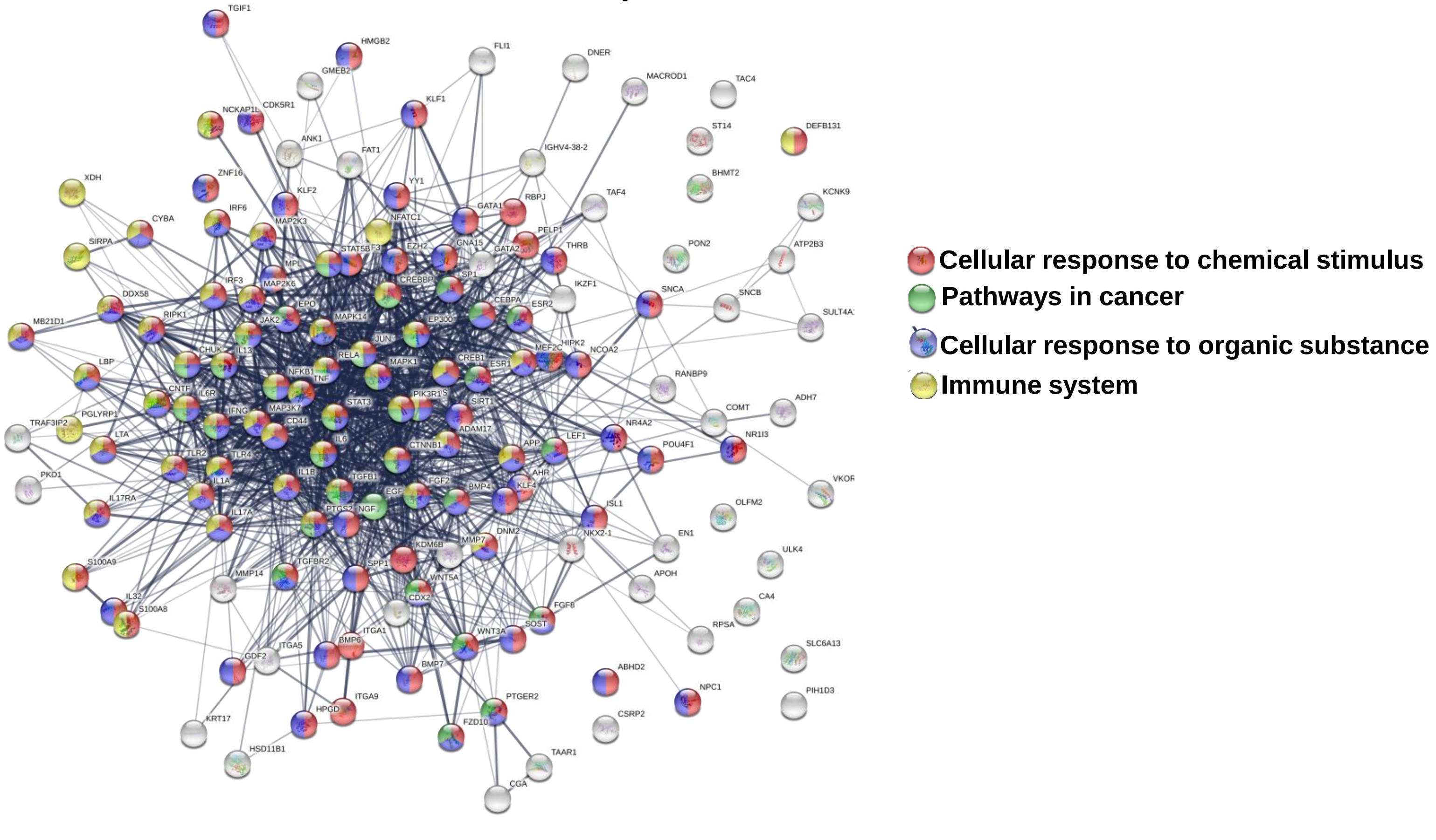

Supplemental Figure 5. Additional fibropapillomatosis inferred transcriptional regulator (ITR) interaction networks. **a, b)** Interaction networks of the top 200 ITRs of established **a)** external tumors and of **b)** lung tumors.

a

Kidney FP versus non-tumored kidney tissue top 200 ITR network (RNA-seq)

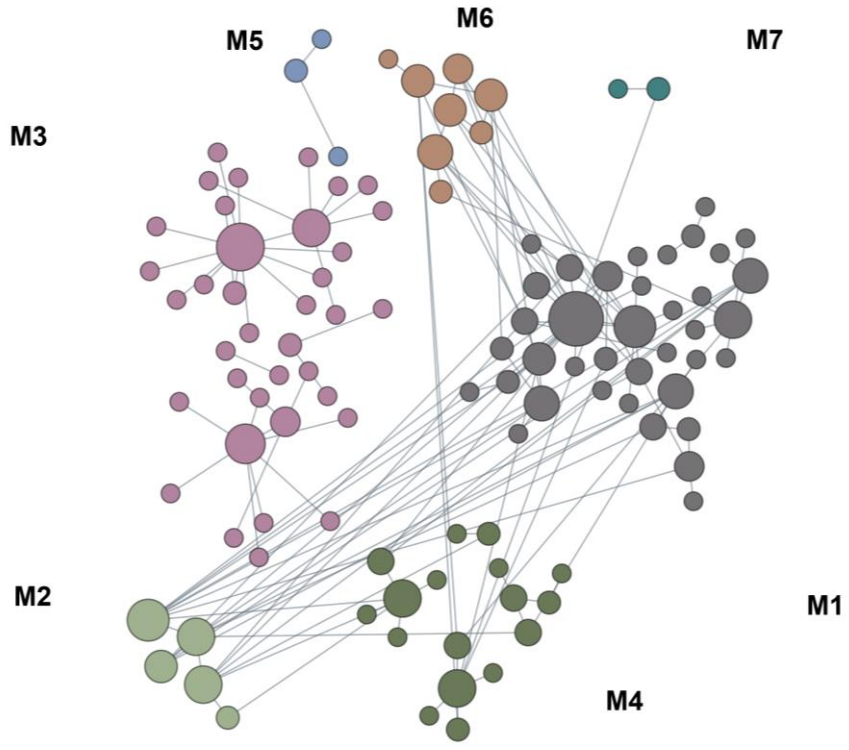

	Kidney Top term	Q value
**M1**	Response to growth factor	<1e-04
**M2**	Cellular response to interleukin 6	<1e-04
**M3**	Epithelium development, embryo development	<1e-04
**M4**	Positive regulation of protein kinase activity	<1e-04
**M5**	Positive regulation of protein chemokine secretion	<1e-04
**M6**	Myeloid leukocyte development	<1e-04
**M7**	Cellular response to growth factor	0.0010

b

New growth external FP versus non-tumored skin top 200 ITR network (RNA-seq)

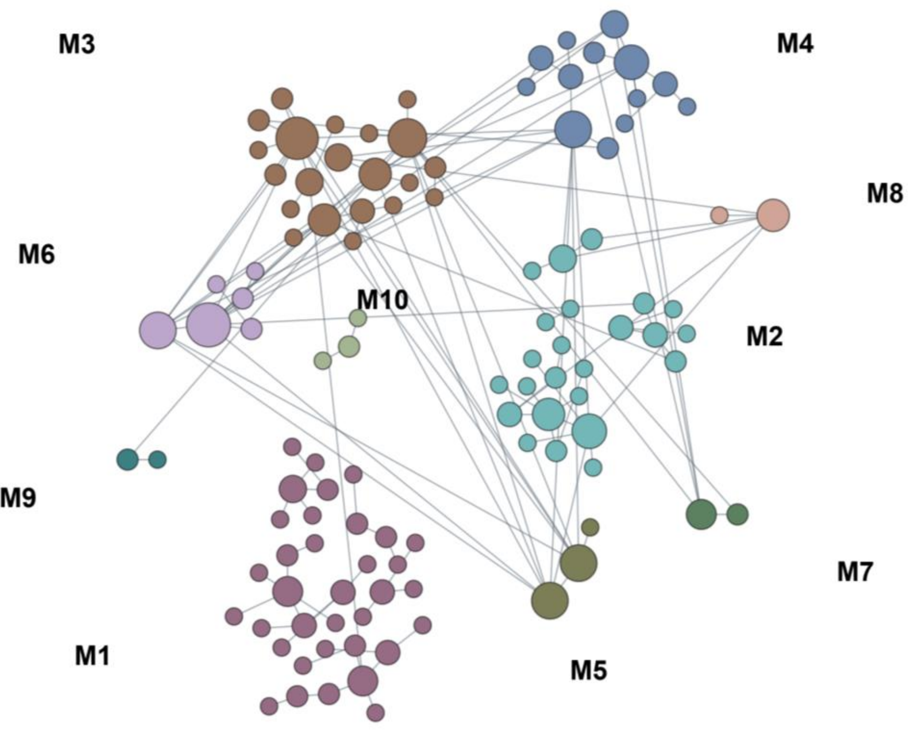

	Top term	Q value
**M1**	Regulation of JAK-STAT cascade	<1e-04
**M2**	Regulation of DNA binding transcription and viral transcription	<1e-04
**M3**	Positive regulation of cytokine production	<1e-04
**M4**	Positive regulation of pri-miRNA transcription	<1e-04
**M5**	Hyaluronan metabolic process	<1e-04
**M6**	Positive regulation of pri-miRNA transcription	0.0001
**M7**	Cellular response to reactive oxygen species	0.0002
**M8**	Cell cycle arrest, negative regulation of cell growth, Ras protein signal transduction	0.0002
**M9**	Positive regulation of protein/peptide secretion	0.0004
**M10**	Regulation of leukocyte cell-cell adhesion	0.0007

c

Regrowth external FP versus non-tumored skin top 200 ITR network (RNA-seq)

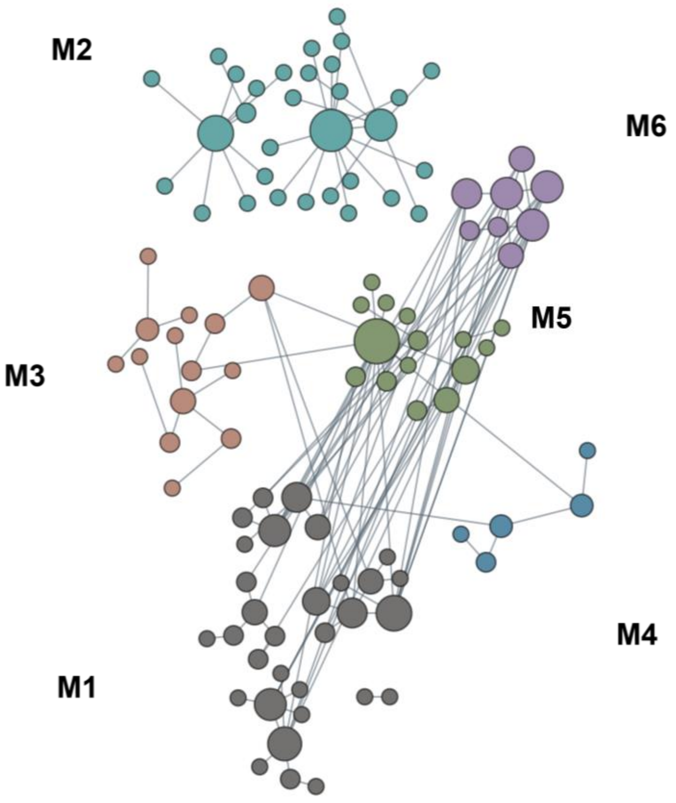

	Top term	Q value
**M1**	Cellular response to cytokine stimulus	<1e-04
**M2**	Cytokine production involved in immune response	<1e-04
**M3**	Positive regulation of cell mobility (EMT)	<1e-04
**M4**	Cellular response to peptide hormone	<1e-04
**M5**	DNA methylation on cytosine	<1e-04
**M6**	Regulation of response to external stimuli (chemotaxis)	0.0001

d

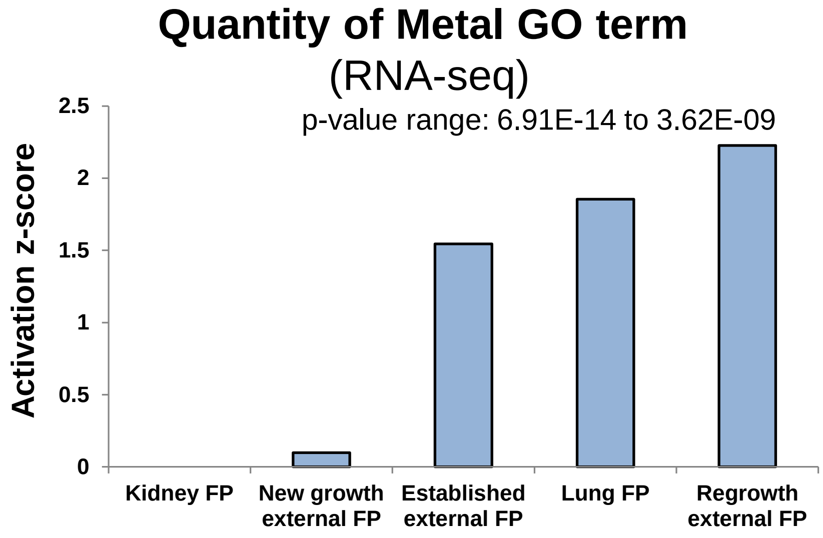

Supplemental Figure 6. Additional fibropapillomatosis inferred transcriptional regulator (ITR) network-based functional module discovery. **a-b)** Network-based functional module discovery of the top 200 ranked ITRs (called by IPA) of **a)** kidney tumors **b)** new growth external tumors and **c)** regrowth external tumors. **d)** Activation z-scores of the 'Quantity of Metal' gene ontology (GO) term associated with transcripts differentially expressed in different types of fibropapillomatosis tumors (kidney FP, lung FP, external FP) when compared to their respective non-tumored tissue sources, as detected by IPA. Note: the kidney FP disease-associated GO analysis did not have the 'Quantity of Metal' GO term called when compared to healthy kidney tissue.

a

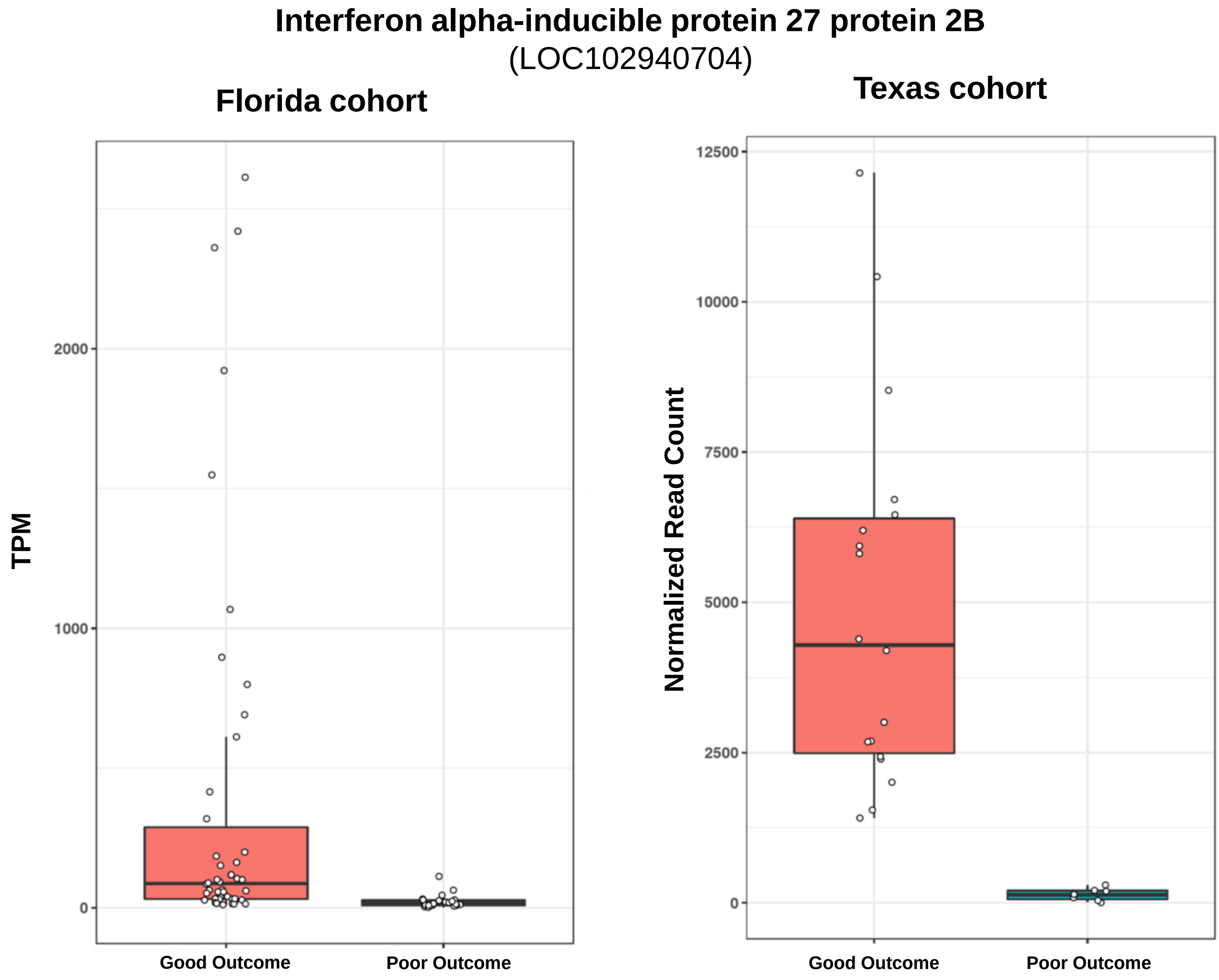

Supplemental Figure 7. Additional outcome expression data. **a)** Expression levels of Interferon alpha-inducible protein 27 protein 2B between tumors of good outcome and poor outcome patients, as detected by RNA-seq. Top: Florida cohort. Bottom: Texas cohort. Florida cohort; N = 69 samples. Per outcome: released (good outcome) = 7 turtles; died/euthanized (poor outcome) = 5 turtles. Texas cohort; N = 25 samples. Per outcome: prolonged rehabilitation (poor outcome) = 2 turtles; short rehabilitation (good outcome) = 1 turtle.
