# Supplementary material for: Molecular characterization of a marine turtle tumor epizootic, profiling external, internal and postsurgical regrowth tumors
Source: Commun Biol. 2021 Feb 1;4:152. doi: 10.1038/s42003-021-01656-7 (PMC7851172; doi:10.1038/s42003-021-01656-7)
Supplement: Supplementary file 3 — Description of Supplementary Files [file 42003_2021_1656_MOESM3_ESM.pdf]

Description of Additional Supplementary Files

File name: Supplementary Data 1

Description: Detailed sample and sequencing read number (raw, trimmed and alignment rate) data table.

File name: Supplementary Data 2

Description: TPM count table data for all 18,442 green turtle genes (CheMyd_1.0 reference assembly), across all RNA-seq samples.

File name: Supplementary Data 3

Description: Patient details from transcriptome profiled (RNA-seq) green turtles (*C. mydas*), number of surgeries, occurrence of regrowth, days in care and outcome.
