# Supplementary material for: Molecular characterization of a marine turtle tumor epizootic, profiling external, internal and postsurgical regrowth tumors
Source: Commun Biol. 2021 Feb 1;4:152. doi: 10.1038/s42003-021-01656-7 (PMC7851172; doi:10.1038/s42003-021-01656-7)
Supplement: Supplementary file 6 — Supplementary Data 3 [file 42003_2021_1656_MOESM6_ESM.docx]

| **ID** | **Number of rounds of surgery** | | **Regrowth occurrence** | **Days in care** | **Outcome** |
| --- | --- | --- | --- | --- | --- |
| ***FL cohort:*** |  |  | |  |  |
| 06-2015-Cm “Major” | 2 | | Yes | 302 days | Released |
| 13-2016-Cm “Emma” | 1 | | No | 148 days | Released |
| 05-2016-Cm “Mean Joe Green” | 6 | | Yes | 523 days | Released |
| 34-2016-Cm “Sebod” | 1 | | No | 93 days | Released |
| 17-2017-Cm “Remi” | 3 | | Yes | 344 days | Released |
| 24-2017-Cm “Rollie” | 3 | | Yes | 241 days | Euthanized |
| 25-2017-Cm  “Tangled” | 4 | | No | 325 days | Released |
| 27-2017-Cm | 0 | | No | 90 days | Euthanized |
| 34-2017-Cm | 0 | | No | 3 days | Euthanized |
| 37-2017-Cm | 0 | | No | 2 days | Euthanized |
| 14-2016-Cm “Apollo” | 3 | | Yes | 169 days | Released |
| 02-2017-Cm “Chrystal” | 3 | | Yes | 209 days | Euthanized |
| 02-2015-Cm “Swoope” | 4 | | No | 253 days | Euthanized |
| ***TX cohort:*** |  | |  |  |  |
| “Beach Bum” | 2 | | No | 140 days | Released |
| “Frostbite” | 3 | | Yes | 257 days | Released |
| “Reveille” | 5 | | Yes | 429 days | Released |
